# First aid facilities in the school settings: Are schools able to manage adequately?

**DOI:** 10.12669/pjms.342.14766

**Published:** 2018

**Authors:** Farhan Muhammad Qureshi, Nadia Khalid, Seema Nigah-e-Mumtaz, Tahira Assad, Khola Noreen

**Affiliations:** 1 Dr. Farhan Muhammad Qureshi, MBBS, MS (Public Health & Health Promotion), Senior Lecturer, Department of Community Health Sciences, Karachi Institute of Medical Sciences (KIMS), Malir Cantt, Karachi, Pakistan; 2 Dr. Nadia Khalid, MBBS. Lecturer, Department of Community Health Sciences, Bahria University Medical & Dental College (BUMDC), Karachi, Pakistan; 3 Prof. Dr. Seema Nigah-e-Mumtaz, MBBS, MPH, M.Phil. Department of Community Health Sciences, Karachi Institute of Medical Sciences (KIMS), Malir Cantt, Karachi, Pakistan; 4 Dr. Tahira Assad, MBBS, M.Phil. Assistant Professor, Department of Pharmacology, Karachi Institute of Medical Sciences (KIMS), Malir Cantt, Karachi, Pakistan; 5 Dr. Khola Noreen, MBBS, M.Phil. Assistant Professor, Department of Community Health Sciences, Bahria University Medical & Dental College (BUMDC), Karachi, Pakistan

**Keywords:** First-aid facility, First aid training, Medical incidents, Schools teachers

## Abstract

**Background and Objective:**

Children spend most of their time in schools and are vulnerable to injuries and mild ailments, hence requiring first-aid care. School teacher can provide immediate first-aid care in the absence of any health professional. This study assesses first-aid facilities within school premises and assessment of teachers on first aid training.

**Methods:**

A cross sectional study was conducted from July-December 2017, participants were full time school teachers of both public and private sectors at both primary and secondary levels, having a minimum of one year experience. Questionnaire was filled on one to one basis by taking oral interview.

**Results:**

Out of 209 teachers, 72.7% were from private sector. Stomachache was the most common medical incident (82.29%) requiring first-aid care in schools. First aid box was available in all schools but its contents were not satisfactory. Sick bay was not found in any school. 68.42% of teachers were not trained in first-aid management because of lack of opportunity, however 56% were willing to enroll in any first aid training and majority (91.38%) considered it essential for their professional life.

**Conclusion:**

First aid facilities at various schools of Karachi and availability of trained teachers who can provide first aid care is unsatisfactory.

## INTRODUCTION

School life is an important part of children’s life, which has a direct impact on their physical and mental health.^1^ Health promotion and prevention are the most neglected part in both policy and practice. Schools can play a significant role in health promotion due to two major reasons; first, the schools offer structured opportunities for learning and second, pupils spend significant amount of their time in schools during which they are involved in a variety of activities including sports and physical exercise.^2^ In developing countries, school health services are often neglected^1^ this directly influences the management of common illnesses such as first-aid care or referral.^3^

Children are vulnerable to injuries and accidents, which may vary from minor injuries to severe accidents resulting in bleeding and fractures, thus the first-aid management becomes as important as taking a child to a medical facility.^4^ Children spend most of their daytime in schools^5^, and are at greater risk of accidents and injuries due to involvement in sports and other extracurricular activities, and hence require first-aid more often than do adults.^1^,^6^ Overall, majority of the injuries among children are directly related to outdoor physical activities, of which 20% occurs during school hours.^7^

First-aid is the treatment given for any injury or sudden illness prior to any professional medical help provided.^8^ The primary objective of first aid is to alleviate suffering, facilitate healing process and minimize damage.^4^ First action taken for management of injuries as a first aid is very crucial as it decides the future course of disease and complication rates.^9^ The knowledge of first aid, when properly applied, can bridge the gap between temporary or permanent injury, rapid recovery, or long-term disability.^10^

Usually, schools do not have trained health care professionals such as doctors or nurses in their premises as a permanent employee. Teachers, as a full time employee are the main care givers and can be the first line protector of children at school. So, it is essential for the teachers to be trained in first aid management. Researchers reported poor or incorrect practices associated with injuries, illnesses and first aid management among school teachers.^4^ Schools need to train their teaching staff and fully equipped with first aid facilities, in order to respond appropriately to the first-aid needs and requirements. They must be able to deal properly with health emergencies both in normal children, and in children with special health care needs.^3^ Teachers also need to keep themselves updated with current first aid guidelines. Results of the school based study in a well-developed country, United States of America; (USA) showed that only 5.4% of the teachers received first-aid training, however, majority of them didn’t updated their first aid training.^1^,^11^

With this background, present study was specially designed to assess the availability of the first-aid facilities within school premises in the schools of Karachi city as well as assessment of teachers for their training on first-aid.

## METHODS

This cross sectional study was conducted from July-December 2017. Participants were school teachers of 11 primary and secondary schools of both public (n=5) and private sector (n=6) of city, Karachi selected after simple random selection. 209 full time teachers having a minimum of one year experience were included. After the informed consent, respondents were given explanation about the purpose of study.

Data was collected by using specially designed structured questionnaire. Each questionnaire was filled on one to one basis by taking an oral interview and confidentiality was ensured. This method of data collection excludes various problems of incomplete filling of the questionnaire, misunderstanding the asked questions and peer influences on filling the questionnaire. Ethical approval from institute was also taken prior to commencement of study.

### Study Tool Questionnaire Development

The questionnaire consisted of two sections. The 1^st^ section contained demographic information of the teachers such as age, gender, academic qualification, teaching experience etc. The 2^nd^ section consisted of a variety of open and closed ended questions regarding first-aid medical facilities in their schools. A total of 15 questions were asked in 2^nd^ section with options such as; got trained in any particular course for handling emergencies, reasons of not trained, common medical incidents happened in schools, contents of first aid kit etc. Data was entered using Microsoft Excel 2010 and analyzed using SPSS version 23. Descriptive statistical measures like frequencies and percentages were calculated.

## RESULTS

Demographic characteristics of the participants are given in Table-I. Out of 209 teachers who participated in this study; majority were females (88.5%), less than 40 years of age (73.7%), 72.2% were employee of private schools. It was estimated that, majority (52.2%) of the teachers had higher education up to the level of master’s degree. The teaching experience ranges between one year to >20 years while 63.6% teachers got experience up to 10 years.

**Table-I:** Demographic characteristics of Participants (n= 209).

Variables		Frequencies (n)	Percentage (%)
Gender	Male	24	11.5
	Female	185	88.5
Age	<40	154	73.7
	>40	55	26.3
Qualification	Intermediate	19	9.1
	Bachelors	81	38.8
	Masters	109	52.2
Type of School	Private	151	72.2
	Government	58	27.8
Teaching experience	b/w 1-10 years	133	63.6
	11-20 years	50	23.9
	>20 years	26	12.5

**Fig.1:**
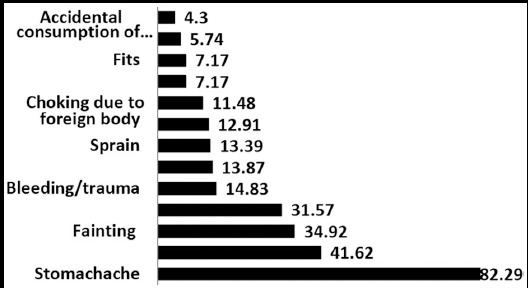
Most commonly reported school medical incidents requiring first-aid treatment (%)

**Fig.2:**
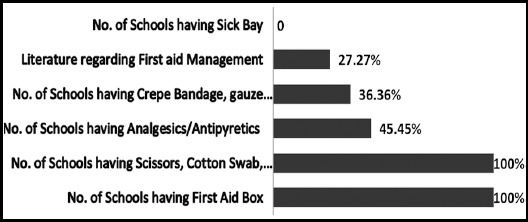
Availability of first aid box in schools and its contents (%).

Fig.2 shows the availability of first-aid box in schools and its contents. Almost all the schools (n=11) had some sort of first aid boxes/kits but none of them were fully equipped. Only scissors, cotton swabs and antiseptic solution (Dettol) were present in all schools. Analgesics/antipyretics syrups or tablets were present in five schools (45.45%), crepe bandages, gauze rolls and adhesive tape were available in the four schools (36.36%) and literature regarding first-aid management was present in three schools (27.27%). However, none of the school had a room specified for the management of sick or ill children (Fig.2).

The responses of teachers regarding first-aid training is shown in Table-II. Only 31.57% teachers had prior training on handling medical emergencies, out of which 24.88% attended a course on first aid management. About 56% of teachers were willing to enroll in first-aid management course while 91.38% were of opinion that training in first-aid care is essential to their professional life.

**Table-II:** Responses of teachers regarding first aid training (n=209).

Questions		Responses	

		Yes	No
Do you have any prior training on handling medical emergencies?		66 (31.57%)	143 (68.42%)
Do you have attended any particular course regarding first-aid?	First Aid Management Course	52 (24.88%)	157 (75.11)
	Basic Life Support (BLS)	10 (4.78%)	199 (95.21%)
	Trauma Care	3 (1.4%)	206 (98.56%)
	Others	1 (0.47%)	208 (99.52%)
What is the reason of not getting trained in first-aid?	Lacks of opportunity	75 (35.88%)	134 (64.11%)
	Lack of time	34 (16.26%)	175 (83.73%)
	Lack of statutory requirement for compulsory training	11 (5.26%)	198 (94.73%)
	Not a part of education	43 (20.57%)	166 (79.42%)
Are you willing to enroll in any training on first-aid management course?		117 (56%)	92 (45%)
Do you think that training in first-aid management is essential to your professional life?		191 (91.38%)	18 (8.61%)

## DISCUSSION

School going children constitute a major part of the gross population of Pakistan and across the world. Due to curiosity, increased mobility and lack of experience, children are vulnerable to injuries and accidents.^12^ Apart from teaching, teachers are a pivotal character for children because they supervise and care for their health related issues. School teachers act as the guardians of the students so they need to be equipped with the adequate knowledge regarding first aid practices.^4^ In the present study, attempt was made to assess the first-aid facilities in schools and preparedness of the teachers and staff to handle medical emergencies.

In this study stomachache was reported to be the most common ailment among school children requiring first aid treatment. Various studies have mentioned stomachache as one of the most commonly occurring symptom in children.^13^,^14^ However, Haugland, in his study, based on an analysis of data from a World Health Organization (WHO) cross-national survey on health behavior in school children, conducted between 1983-1994 reported abdominal pains as the most common symptom in children.^15^

It was found that all 11 schools (100%) visited had a first-aid box. This is in contrast to the studies conducted in Manglore, India;^16^ Chandigarh, India^3^and Mysore, India^4^ where only 55.6%, 62% and 25% of schools had first box respectively. In a study conducted in Ireland, researchers revealed that 35-81 % of schools in Ireland had equipment to deal with injury.^11^ They also observed that not a single school had a fully equipped first aid kits/box.^3^ Regarding contents of first aid box, although all the schools in this study had first aid box but it is very disappointing that none of the first aid box was fully equipped. When enquired about first aid medicines, it was revealed that the only medicine was paracetamol syrup or tablets which they use as a painkiller or for treatment of fever and antiseptic solution or ointment. Similar findings were observed in a study conducted in Manglore, South India.^16^ However, in another study conducted in Chandigarh, India researcher mentioned paracetamol and antiseptic ointment was available in only 16% and 22% of schools respectively.^3^

In the present study,31.97%teachers had received previous some sort of training in first aid management, however (24.88%) attended specific first-aid management course. In comparison, studies conducted in Vadodara, India;^17^ Mysore, India;^4^ Manglore, India;^16^ Western Turkey;^18^ and China,^19^about 3, 16, 47, 31 and 62% of the teachers were trained in first aid management respectively. Despite the fact that only one third of teachers had some sort of training in first aid management in our study, results showed that teachers had positive attitudes about the importance of first aid and learning first aid management course as common reasons highlighted for not obtaining training in first-aid were the lack of opportunity and time. This was reported by 52.14% of the teachers who had not received any previous training. The lack of compulsory first aid training as a part of education or during teacher training was also a reason given by 26% teachers. Most of them (56%) were willing to enroll in first aid management course and majority (91.38%) of them considered it essential for their professional life. Similar observations were found in a study conducted in Ireland^11^ United State of America^20^ where teachers agreed that first aid training should be included in curriculum or teacher training programs.^20^

Yurumez in his study conducted in Turkey reported that teachers’ training in first-aid was not up to the mark and majority of teachers reported a need for attending training on first-aid.^18^ In a study conducted in Egypt, author reported that inadequate knowledge of school teachers regarding first-aid management was due to lack of training in the curriculum of the specific education.^21^ The unsatisfactory knowledge in the present study can be attributed to the fact that very little importance is given by school management and functionaries towards training of school teachers on first aid. Therefore, it seems a pressing necessity to strengthen school health services through full participation of teachers and educators, providing them training sessions and update them by regularly interacting with them. The current concept of the school health programs bring together experts form health related sectors along with the parents and professionals from the education to provide a comprehensive primary health care service to children.^22^ Here the researcher would highlight the importance of school health care services by formulation of school health care team which is consisted of a medical officer, a trained nurse, a lady health visitor (LHV), a dispenser, an aaya and a sweeper.

### Limitations

The study did not assess the knowledge and skills of teachers regarding handling of medical emergencies. Evaluation of practical skills and knowledge would have further helped in understanding the problems faced by the children rendering first-aid emergencies.

### Strengths

No similar studies regarding preparedness of schools to deal with ailments and injuries in children and the availability of first-aid facilities have been done in Pakistan before. The study revealed that first-aid measures need to be improved in schools. This is possible by introducing formal first-aid training in the teaching curriculum. Also the school health care team must be ensured in all educational institutions.

## CONCLUSION

This study identified the deficient first-aid facilities in various schools of Karachi as well as trained teachers regarding first-aid care. It is recommended that school health services need to ensure the provision of school health care team, fully equipped first aid box/kit in schools along with a separate sick room to handle medical cases. Schools management must design programs to train the school teachers on first aid management and incorporate the same in regular school health appraisal. These measures will serve to make schools a safer environment for children.

### Author`s Contribution

**FMQ:** Conceived the main research idea & developed the study design, developed the study tool i.e. Questionnaire, literature Survey, Manuscript Write up and bibliography.

**NK:** Data collection and Analysis, interpretation of results.

**SM:** Research Supervisor, Critical checking and analysis of the results, final approval of the manuscript.

**TA and KN:** Helped in data collection, data entry in Excel, analysis and Tabulation, Final proof read for any grammatical or language errors.
